# Regeneration of Soft Tissues Is Promoted by MMP1 Treatment after Digit Amputation in Mice

**DOI:** 10.1371/journal.pone.0059105

**Published:** 2013-03-18

**Authors:** Xiaodong Mu, Ian Bellayr, Haiying Pan, Yohan Choi, Yong Li

**Affiliations:** 1 The Laboratory of Molecular Pathology, Stem Cell Research Center, Children’s Hospital of UPMC, Pittsburgh, Pennsylvania, United States of America; 2 Department of Orthopaedic Surgery, University of Pittsburgh, School of Medicine Pittsburgh, Pennsylvania, United States of America; 3 Department of Bioengineering, University of Pittsburgh, Pennsylvania, United States of America; 4 Department of Pediatric Surgery, University of Texas Medical School at Houston, Houston, Texas, United States of America; 5 Center for Stem Cell Research and Regenerative Medicine, University of Texas Health Science Center at Houston, Houston, Texas, United States of America; University of Kansas Medical Center, United States of America

## Abstract

The ratio of matrix metalloproteinases (MMPs) to the tissue inhibitors of metalloproteinases (TIMPs) in wounded tissues strictly control the protease activity of MMPs, and therefore regulate the progress of wound closure, tissue regeneration and scar formation. Some amphibians (*i.e*. axolotl/newt) demonstrate complete regeneration of missing or wounded digits and even limbs; MMPs play a critical role during amphibian regeneration. Conversely, mammalian wound healing re-establishes tissue integrity, but at the expense of scar tissue formation. The differences between amphibian regeneration and mammalian wound healing can be attributed to the greater ratio of MMPs to TIMPs in amphibian tissue. Previous studies have demonstrated the ability of MMP1 to effectively promote skeletal muscle regeneration by favoring extracellular matrix (ECM) remodeling to enhance cell proliferation and migration. In this study, MMP1 was administered to the digits amputated at the mid-second phalanx of adult mice to observe its effect on digit regeneration. Results indicated that the regeneration of soft tissue and the rate of wound closure were significantly improved by MMP1 administration, but the elongation of the skeletal tissue was insignificantly affected. During digit regeneration, more mutipotent progenitor cells, capillary vasculature and neuromuscular-related tissues were observed in MMP1 treated tissues; moreover, there was less fibrotic tissue formed in treated digits. In summary, MMP1 was found to be effective in promoting wound healing in amputated digits of adult mice.

## Introduction

MMPs are activated during the wound healing process and are important regulators of ECM remodeling and tissue regeneration [1]–[4]. The ratio of MMPs/TIMPs is suggested to be a critical determinant of the transition from scarless healing to wound healing with scars, and the higher ratio of MMP to TIMP expression could be associated with scarless healing in amphibians and fetal mammals [5]–[10]. For example, it has been reported that skin wound healing of fetal rats transits from scarless repair to scarring repair between days 16.5 (E16) and 18.5 (E18) of gestation, with scarless wounds having greater MMP (i.e., MMP1, MMP2 and MMP14) relative to TIMP (i.e., TIMP-1 and TIMP-3) expression than scarring wounds [5]. Similarly, a greater MMP to TIMP ratio of MMP2 and MMP9 was detected in the MRL/MpJ strain of mice, in which through-and-through ear hole punches are able to heal without the formation of scars within a few weeks [11].

MMP1 expression is suggested to be controlled by cell-collagen interactions [12], [13], and presumably aids the migration of tissue progenitor cells by degrading type I and III collagen at the site of injury [4], [12]. Recent studies performed both *in vitro* and *in vivo* have shown the beneficial impact of MMP1 administration on muscle healing [14]–[17]. For example, MMP1 treatment of muscle cells *in vitro* was shown to increase the migration and myogenic differentiation capacities of the cells [10], [14]; transplantation of C2C12 myoblasts in combination with MMP1 into skeletal muscle of MDX/SCID mice, or injection of MMP1 alone to a site of injury showed improved cell migration and increased myofiber formation, as well as reduced fibrotic tissue formation [14].

The regeneration of digit tips, the digit, and even the whole limb has been intensively studied in the field of regenerative medicine. For newts or fetal mice, the amputated limbs or digit tips were observed to fully regenerate, which does not normally occur in mammalian wound healing [18], [19]. MMPs have been found to be up-regulated very early after digit or limb amputation and are required for this regeneration process [20], [21]. The healing of a wound or wound closure is the first step in digit or limb regeneration [21]. It was proposed that MMPs contribute to the digit or limb regeneration by promoting ECM degradation and the formation of the wound epidermis, which is formed by the migration of epidermal cells to the perimeter of the amputation surface and is necessary for wound closure [18], [20], [21].

Although numerous studies have shown that amputation of a digit tip distal to the mid-third phalanx resulted in almost complete regeneration, amputation proximal to this region does not support regeneration without assistance from exogenous factors. Due to the numerous beneficial effects of MMP1, we proposed to investigate the effect of MMP1 treatment in improving the wound healing process and reducing scar formation, *e.g*. scarless healing, after digit amputation. In this study, the middle digits of both hind feet of mice were amputated through the mid-second phalanxes [22], [23]. MMP1 was then administrated at the amputated digits every 4 days after the amputation to observe whether increased MMP activity can affect the process of wound healing (wound closure, vascularization, and innervation), digit regeneration and scar formation in the digits. In this study we found that application of MMP1 to amputated digits promoted faster wound closure and regeneration of soft tissues with decreased scar tissue formation.

## Materials and Methods

### Digit Amputation

**Figure 1:**
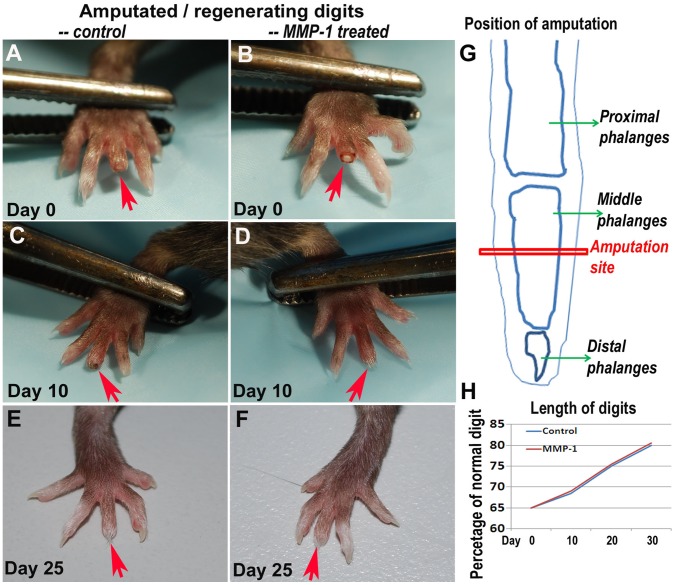
Process of digit amputation and regeneration. Digits were amputated at the central position of the middle phalanges (***A–B***, day 0) (n = 4 for each group). At the early stage of digit regeneration, the wound closure was shown to be faster in MMP1 treated digits (***C–D*** *,* day 10). However, during the whole process of digit regeneration, there was no significant difference in the length of the regenerating digits (***C–D*** *,* day 10; ***E–F*** *,* day 25). The site of the amputations is demonstrated (***G***). The lengths of the regenerating digits from day 0 to day 30 after amputation are also plotted as the percentage of the length of normal digits (***H***).

**Figure 2:**
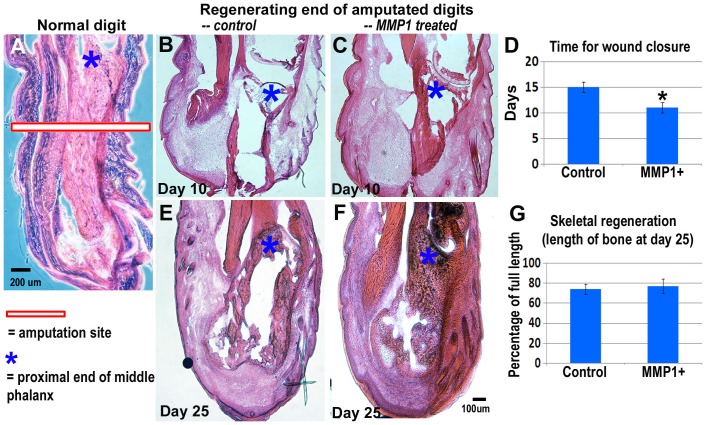
MMP1 treatment accelerated wound closure and healing of soft tissue but not the hard tissue. With hematoxylin and eosin (H&E) staining of tissue sections, soft tissues and bones can be observed in normal digits (**A**) and regenerating digits 10 days (***B&C***) or 25 days after amputation (***E&F***). MMP1 treated digits showed improved regeneration of soft tissues (faster wound closure) (***B–D***), but not significant improvement in the growth or elongation (***E–G***). N = 4 for each group; *p<0.05 was considered as significant.

This study was carried out in accordance with the recommendations in the Guide for the Care and Use of Laboratory Animals of the NIH. The protocol was approved by the Institutional Animal Care and Use Committee (IACUC) of University of Pittsburgh Medical Center (Protocol 0904641), and the Animal Welfare Committee of the University of Texas Health Science Center at Houston (Protocol 12–051). All surgery was performed under isoflurane anesthesia, and all efforts were made to minimize suffering. An inbred strain of mice (C57BL/6J, male, 5 weeks of age, Jackson lab, Bar Harbor, Maine) was used in this study. After being cleaned with 70% alcohol, the middle digits of both hind feet of mice were amputated by blades, through the middle phalanx bones, as shown in ***Fig. 1A–B&G*** and ***Fig. 2A*** *.* The wounded digits in both legs were cleaned with water and treated with antibiotics to avoid bacterial infection.

### MMP1 Administration

Within the first 10 days after amputation, MMP1 was applied directly to the exposed area of the amputated digit of the left legs (300 ng of MMP1 in 3 µl of H_2_O), and injected with a micro-syringe 2–3 mm away from the edge of the severed tip (300 ng of MMP1 in 3 µl of H_2_O). From day 10 to day 25 after amputation (after wound closure), MMP1 was injected only, which was repeated every 4 days. The amputated digits on the right leg were administered PBS to serve as a control.

### Immunohistochemical Analysis of Tissue Sections

Serial 10-µm cryostat sections of regenerating digits (day 10 and day 25) were prepared using standard techniques. For immunohistochemistry, the slides were fixed with formalin (4%) for 5 minutes, and then blocked with horse serum (10%) for 1 hour. Primary antibodies used in the study included: CD31 (BD Biosciences #553370) Utrophin (Santa Cruz #7459), Neural cell adhesion molecule (NCAM) (Millipore MAB310) and dystrophin (Abcam #15277 Cambridge, MA) and were applied to the slides at a 1∶200 dilution for 3 hours at room temperature (RT). Secondary antibodies, IgG (Alexa Fluor 488 or 594; Invitrogen; 1∶400), were incubated with sections for 45 minutes at RT. Negative controls were performed concurrently with all immunohistochemical staining. The nuclei of the sections were revealed using 4′,6′-diamidino-2-phenylindole dihydrochloride (DAPI). Fluorescence microscopy (Leica Microsystems Inc., Bannockburn, IL) was used to examine all of the immunofluorescence results and capture photographic images.

### Trichrome Staining

To detect the amount of fibrosis in the regenerated digits 25 days after amputation, sections of digits were washed in deionized water and stained with a Masson Modified IMEB Trichrome Stain Kit (IMEB Inc, San Marcos, California) according to the manufacturer’s specifications. This technique distinguishes cells from the surrounding connective tissue, generally staining cells red and extracellular collagen blue. It was previously validated through immunohistochemistry as an accurate technique for evaluating fibrotic tissue within soft tissue [24]–[26]. Images were analyzed using Northern Eclipse image analysis software (Empix Imaging) to measure the percent area of collagen (blue staining tissue) within the injury zone. Color threshold levels within the software program were set to isolate the blue staining regions and calculate the area of that region that corresponded to the area of fibrosis. This value was expressed as a percentage of the entire cross-sectional area of the muscle section.

### Statistical Analysis

Tissue sections from 4 identically treated mice for each group were used to generate the data; data pooled for statistical analysis was analyzed based on 3 to 5 pictures of each level of tissue sections. Northern Eclipse image analysis software was used for quantification of all analyses. All of the results are expressed as the mean ± standard error (SE). The differences between means were considered statistically significant if *Ρ value* <0.05. The Mann-Whitney *U* test was used to compare the differences between different groups of tissue sections.

## Results

### MMP1 Treatment Accelerated Wound Closure and Healing of Soft Tissue in the Amputated Digits, but does not Affect the Elongation of the Skeletal Tissue

The middle digits of both hind feet of mice were amputated and MMP1 was administrated to the digits on the left, with the digits on the right serving as the controls. The pictures of the digits were taken on day 0, 10 and 25 after the amputation (***Fig. 1A–H***). Our results show that on both 10 days (***Fig. 1C–D***) and 25 days (***Fig. 1E–F***) after amputation, there was no significant difference in length between MMP1 treated and non-treated groups (***Fig. 1H***); however, the results of hematoxylin and eosin (H&E) staining (***Fig. 2***) indicated that MMP1 treatment of digit tips accelerated the soft tissue wound healing compared to non-treated control digits 10 days after amputation **(** ***Fig. 2B–C***). Wound closure of MMP1 treated digits was shown to be almost complete at day 10, but was incomplete for non-treated digits (***Fig. 2B–D*** **)**. At 25 days after amputation wound closure was also complete in the untreated digits; the lengths of treated and untreated digits continued to show no significant difference at this time point (***Fig. 2E–G***). These results indicate that the wound healing of soft tissues but not the bones was obviously improved with MMP1 administration, suggesting that although MMP1 treatment cannot fully regenerate an amputated digit to its original size, it did, however, have a positive impact on the healing of soft tissues of digits.

### MMP1 Treatment Generated More CD31 Positive Capillary Vasculature 10 Days and 25 Days after Amputation

**Figure 3:**
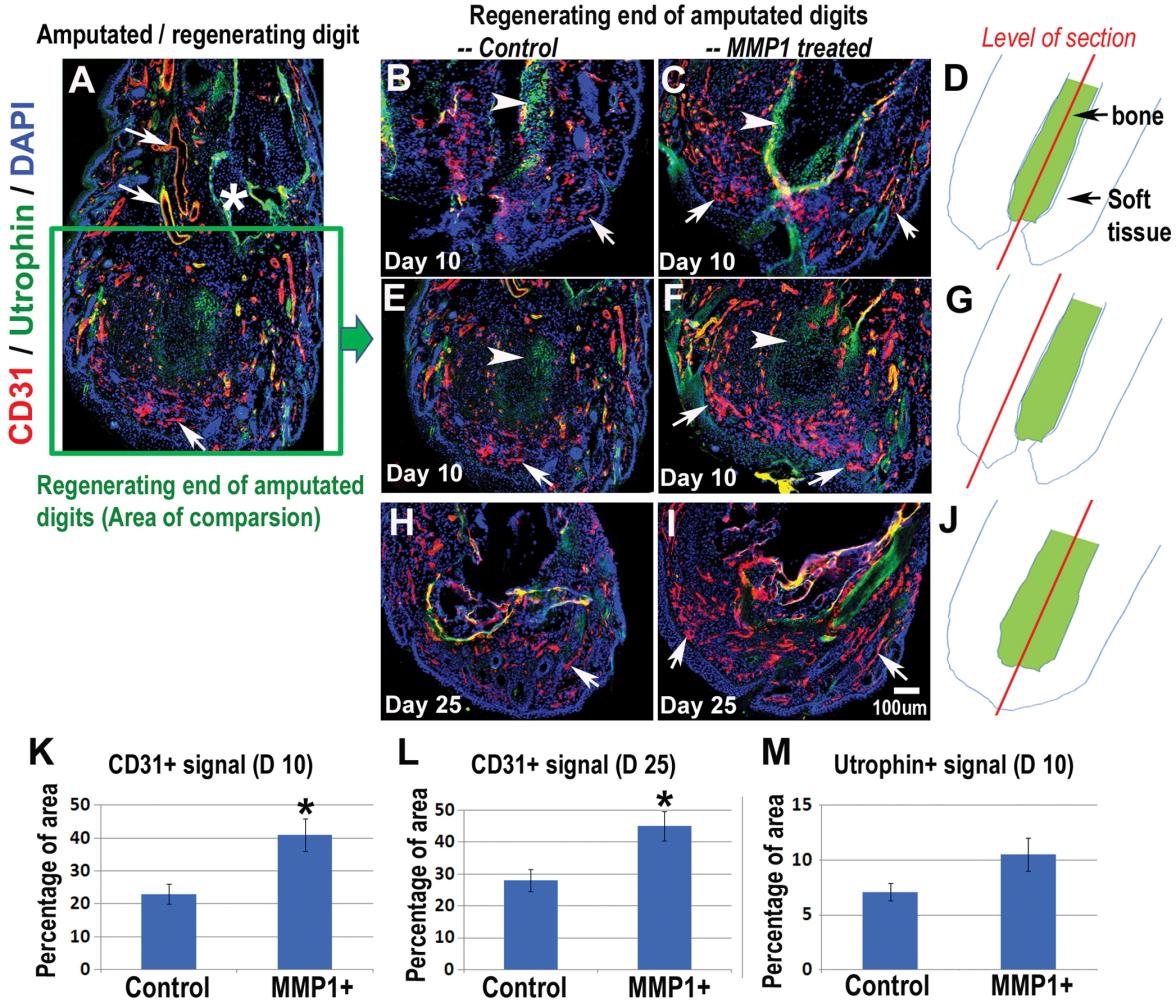
MMP1 treatment improved angiogenesis and re-vascularization in the amputated digits. The localization of CD31 and utrophin proteins in the regenerating digits (***A***) was compared between MMP1 treated and non-treated digits (***B–C***, day 10, sections with bone; ***E–F***, day 10, sections without bone; ***H–I***, day 25, sections with bone). “*” in image **A** indicates the proximal end of middle phalanx. Arrows (red): CD31 positive blood vessels or capillary vasculature; arrowheads (green): utrophin positive cells; DAPI (blue): present cell nucleus. The level/position of sections in each comparison is demonstrated (***D*** *,* ***G &J***). The statistical analysis of CD31 positive signal (***K*** *,* day10; ***L*** *,* day 25), and utrophin positive signal (***M***, day 10) in the amputated digits are also shown. N = 4 for each group; *p<0.05 was considered as significant.

Angiogenesis or re-vascularization is an important natural process during wound healing. It was previously shown that vascular supplies differ in regenerating and non-regenerating amputated rodent digits [22], and revascularization in the amputated digit is supposed to be crucial for improved digit regeneration. Also, an *in vitro* study showed that MMP1 was able to promote vascular tube formation on type I collagen, which is the an important component of ECM [27]. Therefore, we postulated that our observation of the accelerated healing of soft tissues may be related with improved revascularization from MMP1 treatment. To verify this postulation, the deposition of CD31 protein in the regenerating end of the amputated digits was analyzed on tissue sections of amputated digits (10 days and 25 days after amputation) (***Fig. 3A***). CD31 has been identified to be specifically expressed in capillary vasculature and mature blood vessels [28]. Our immunohistochemical results showed an increased density of CD31 positive capillary vasculature at the regenerating end of MMP1 treated digits, both 10 days (***Fig. 3B–G&K***, arrows, red) and 25 days (***Fig*** **. *3H–J&L*** **,** arrows, red) after amputation. The improved revascularization of MMP1 treated digits may contribute to the accelerated wound closure and wound healing of soft tissues.

In this experiment, utrophin was also co-stained to localize mature blood vessels (***Fig. 3A*** ***,*** arrowheads, green) and skeletal muscle-related cells [29], [30]. Utrophin is also found to be present in the regenerating muscle [30] and distal region of the developing digits, including tendons, tendon primordial, and other pre-skeletal masses [31]. Although there appeared to be a trend of higher utrophin expression with MMP1 treatment, results at 10 days after amputation displayed no significant difference in utrophin deposition between MMP1 treated and untreated digits (***Fig. 3B–G&M***, arrowheads, green).

### MMP1 Treatment Generated More NCAM Positive Structure (Peripheral Nerve Fibers and Neuromuscular Junctions) 25 Days after Amputation

**Figure 4:**
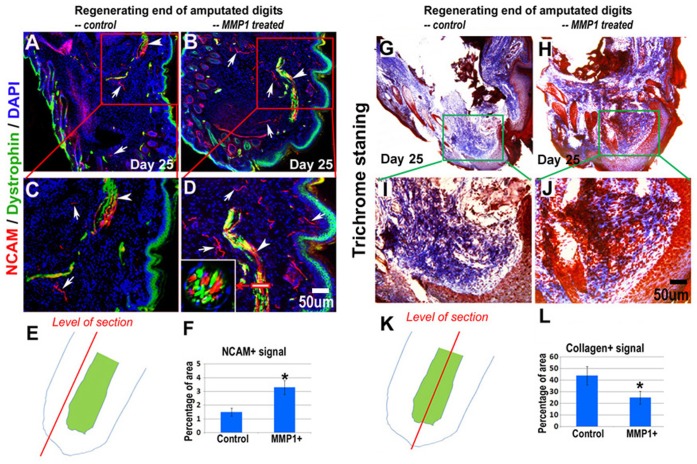
MMP1 treatment improved nerve regeneration and reduced fibrosis formation in the amputated digits. The localization of NCAM and dystrophin proteins in regenerating digits was compared between MMP1 treated and non-treated digits at day 25 (***A–D***). The sub-image in **D** shows the cross-section view of the nerve-related structure (***D*** **,** marked area with red squared line). Arrows: NCAM positive peripheral nerves (red); arrowheads: NCAM positive nerve-related structure (yellow). Dystrophin expression is shown in green and cell nucleus blue (DAPI). The level/position of sections in this comparison is demonstrated (**E**). NCAM expression in the amputated digits was significantly higher in MMP1 treated digits (**F**). To compare fibrosis formation in the differentially treated digits, trichrome staining was conducted with tissue sections of non-treated digits (***G&I***) and MMP1 treated digits (***H&J***). The level/position of sections in this comparison is demonstrated (**K**) and the quantification of fibrotic structure (collagen deposition in ECM) at day 25 shown (**L**). N = 4 for each group; *p<0.05 was considered as significant.

Innervation or nerve regeneration was also found to be crucial for limb regeneration of salamanders [32], and limb regeneration occurs only if there is simultaneous nerve regeneration. To examine the potential effect of MMP1 on nerve regeneration in the amputated digits of mice, the deposition of NCAM (CD56) was analyzed, which is present in peripheral nerve fibers and neuromuscular junctions [33], [34] and abundantly secreted during nerve regeneration [35], [36]. Dystrophin was co-stained to visualize the muscle cells in juxtaposition to the neuromuscular junctions [37]–[40]. Twenty-five days following digit amputation, MMP1 treated digits contained more NCAM positive structures within the peripheral nerve fibers and neuromuscular junctions (***Fig. 4A–D*** *,* arrowheads, red). Based on this observation, the increase in the presence of NCAM positive structures in amputated digits that received MMP1 treatment (***Fig. 4F***) suggests improvement in nerve regeneration or neuromuscular junctions, which may aid in functional recovery.

### MMP1 Treatment Generated Reduced Fibrotic Scar Tissue 25 Days after Amputation

The leading complication of tissue regeneration from injuries or disease has been the formation of fibrotic tissue, which results in an excessive amount of fibrous connective tissue deposited into the ECM space of damaged tissues [41]–[43]. Severely fibrotic tissue will develop chronic healing problems resulting in tissue/organ dysfunction. Previously, MMP1 has been shown to effectively repress fibrosis by digesting collagens types I and III, which are the main constituents of the fibrotic tissue [16], [44]. However, in the wounded tissues, the collagenase activity of MMP1 is often repressed by up-regulated cytokines like TGF-β1, a key factor in the activation of the pro-fibrotic cascade that occurs following the injuries and diseases [25], [43], [45]–[47]. In the regenerating digits, it was investigated whether MMP1 treatment of the digits form less fibrotic tissue 25 days after amputation in comparison to non-treated digits. The results of trichrome staining showed less deposition of extracellular collagen within MMP1 treated digits 25 days after amputation compared to MMP1 non-treated digits (***Fig. 4G–L***). This result indicates the positive effect of MMP1 in repressing fibrosis formation during digit regeneration.

### MMP1 Treatment Increased Sca-1 Positive Progenitor Cells

**Figure 5:**
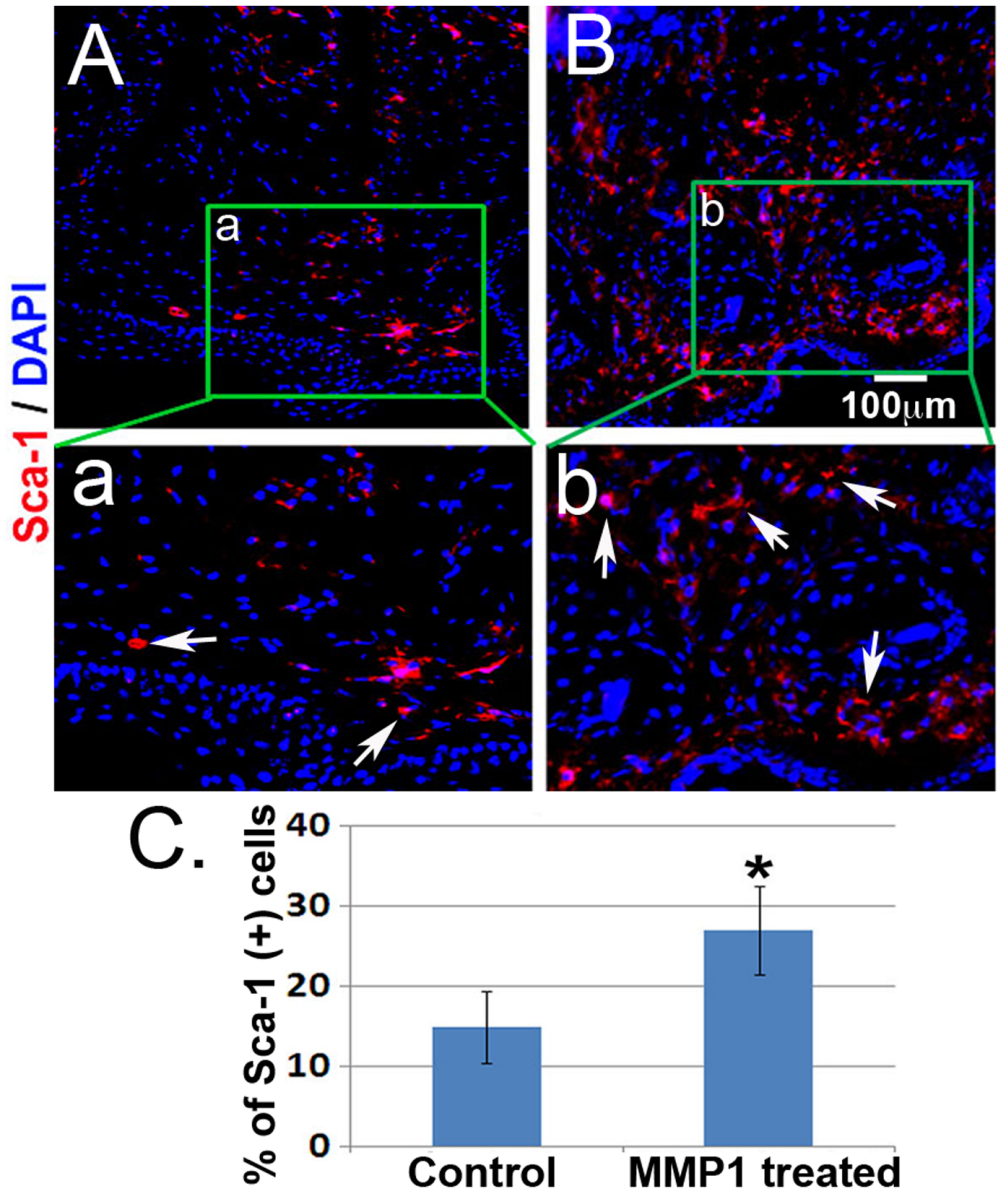
MMP1 treatment enhanced stem cell population. The localization of Sca-1 expressive cells at 10 days after amputation is compared between untreated (***Aa***) and MMP1 treated (***Bb***) regenerating digits. The number of expression of Sca-1 positive cells was enriched in MMP1 treated digits compared to non-treated digits, respectively (***C***). Arrows (red): Sca-1 positive cells; DAPI staining (blue): indicated cells’ nucleus. N = 4 for each group; *p<0.05 was considered as significant.

Sca-1 (Stem Cell Antigen-1) is a member of the Ly-6 family and is expressed on multipotent hematopoietic stems cells as well as several non-hematopoietic progenitor cells, including myogenic progenitor cells. The localization of Sca-1 positive cells 10 days after amputation was compared between untreated (***Fig. 5A*** ***a***) and MMP1 treated (***Fig. 5B*** ***b***) regenerating digits. Our results indicated the expression of Sca-1 positive cells (***Fig. 5A–B*** ***,*** arrows, red) was significantly enriched in MMP1 treated tissues compared to non-treated digits (***Fig. 5C***).

## Discussion

The natural regeneration of a missing digit in adult mammals had been considered unachievable because of the modified immune response, gene expression profile, or mechanisms of wound healing when compared to digit regeneration in amphibians or fetal mammals. Understanding these different mechanisms of wound healing will be important for future studies to induce regeneration of digits and even limbs in adult mammals. MMPs have been recognized to play critical roles in digit regeneration of amphibians and fetal mammals, with a higher MMPs/TIMPs ratio being often observed during the processes [2], [3]. In this study, by applying exogenous MMP1 in the amputated digits of adult mice, we observed that the elevated ratio of MMPs/TIMPs improved tissue regeneration of the amputated digits. Our results demonstrate that wound closure and healing of the soft tissues were greatly improved in MMP1 treated digits, although the healing of skeletal tissue and digit elongation was not significantly improved. MMP1 treatment resulted in the increased formation of capillary blood vessels, peripheral nerve fibers and neuromuscular junctions, as well as decreased formation of fibrotic scar tissues in the amputated digits.

Activation of MMPs was suggested to be critical during the process of limb regeneration of newts and digit tip regeneration of neonatal mice [20], [48]. Our previous studies have indicated the essential role of MMP1 during the muscle healing process and have demonstrated that MMP1 injection into healthy tissue does not induce damage [14]–[17]. Various types of MMPs were also found to be able to promote angiogenesis/revascularization and nerve regeneration [49]–[52], which would be beneficial to the functional recovery of regenerating digits. The overall positive role of MMP1 in promoting soft tissue healing may be related to up-regulating inflammatory proteins that assist in removing tissue debris [53], [54], which in turn improves cell migration of various types (i.e., inflammation cells and muscle progenitor cells) [4], [10], [14], accelerates myogenic differentiation of muscle progenitor cells [4], [14], and reduces fibrosis formation by repressing different fibrotic factors during ECM turnover [4], [15], [16].

Wound closure occurs rapidly in regenerating amphibians; however, it is slow in non-regenerating mammals. MMP1 treatment improved the wound closure time, which is the initial step of both wound healing and regeneration (***Fig. 2B–D***). Conversely, mice with a mutation in collagen I that rendered it insensitive to cleavage by MMP1 demonstrated impaired tissue remodeling and severely delayed wound healing [55]–[57]. Thus MMP1 treatment can be seen as an element in the bridge to transit from non-regenerating scar formation to full regeneration.

The observations of increased utrophin positive cells in healing digits may suggest a possible role of the protein in digit regeneration, as it is also involved in embryonic digit development [31]. The utrophin gene was previously found to be transcriptionally up-regulated in the distal region of the developing digits including tendons, tendon primordial, and other pre-skeletal masses [31]. Our results indicate that utrophin deposition was detected in both MMP1 treated and non-treated amputated digits (***Fig. 3B–G&M***, arrowheads, green); however, there was no statistical difference between treated and untreated digits. Further study is needed to address utrophin’s role in the digit regeneration after amputation injury. Although the skeletal tissue in amputated digits could not regenerate fully to its original architecture and no significant differences were observed in the elongation of skeletal tissue with or without MMP1 treatment (***Fig. 2E–F***), these results suggest that MMP1 has the ability to promote soft tissue regeneration. More detailed mechanisms and potential functional recoveries are under investigation by our research team. Bone regrowth was demonstrated in neonatal amputation models via addition of BMP2 or BMP7 [58]–[61]. Whether a combination of MMP1 with BMP would induce a similar effect in adult mice remains to be determined. Additionally, it is possible that bone regrowth may potentially result in digit elongation to the original length. Future research will focus on bone and cartilage regrowth after digit amputation. In conclusion, our results indicate that the activation of MMPs in the amputated digits of adult mammals promote regeneration of soft tissues with little fibrous scar tissues, but does not affect the digit bones.
